# Guillain-Barré Syndrome Presenting With Masticatory Disturbance and Reduction in Bite Force

**DOI:** 10.7759/cureus.47174

**Published:** 2023-10-17

**Authors:** Kazuya Yoshida

**Affiliations:** 1 Department of Oral and Maxillofacial Surgery, National Hospital Organization, Kyoto Medical Center, Kyoto, JPN

**Keywords:** occlusal force, mastication training, rehabilitation, masseter muscle, masticatory disturbance, bite force, guillain-barré syndrome

## Abstract

Guillain-Barré syndrome is a rare, rapidly progressive, and potentially fatal immune-mediated neuropathy. A 17-year-old male patient with a fever of 38°C developed masticatory impairment a few days later. Although the fever resolved after one week, the restricted mouth opening and decreased bite force persisted. Thirty-five days after disease onset, the patient was referred to our clinic and reported severe masticatory dysfunction due to a significant reduction in maximum bite force (left: 0 N, right: 12.7 N). His maximal mouth opening was 24 mm without jaw deviation. An electrophysiological study revealed Guillain-Barré syndrome (acute motor axonal neuropathy). The patient was closely monitored as he underwent rehabilitation comprising mouth opening and mastication training. The patient’s bite force and masticatory impairment due to the weakness of the muscles of mastication gradually improved. At one year and five months after disease onset, the masticatory impairment had fully resolved. His maximum bite force two years after disease onset was 158.3 N on the left side and 172.2 N on the right side.

## Introduction

Guillain-Barré syndrome (GBS) is characterized by the presence of acute areflexic paralysis and albumin-cytological dissociation. The disease was first described in 1916 [1]. GBS is a rare, rapidly progressive, potentially life-threatening, immune-mediated disease of peripheral nerves and nerve roots [2], with an annual global incidence of approximately 1-2 per 100,000 person-years [3]. GBS is often preceded by an infection or other immune stimulation, which induces an aberrant autoimmune response that targets peripheral nerves and their spinal roots [4,5]. Two-thirds of GBS cases are preceded by symptoms of upper respiratory tract infection or diarrhea [6]. Rapidly progressive bilateral weakness is the key presenting symptom in most patients with GBS [7]. Patients with GBS typically present with weakness and sensory signs in the lower limbs that progress to the upper limbs and cranial muscles [2]. The disease can progress rapidly, and most patients with GBS reach their maximum disability within two weeks [2]. Mechanical ventilation is required in up to 30% of patients with GBS; moreover, the disease has a mortality rate of 5-12% [5].

The muscles of mastication can often become hyperactive due to several etiologies, such as bruxism, temporomandibular disorder, or oromandibular dystonia [7]; however, acute cases of progressive weakness of masticatory muscles are very rarely seen in the outpatient clinic of an oral and maxillofacial surgery department. GBS is the most frequent cause of subacute neuromuscular weakness worldwide [6]. Here, we report a case of GBS in a patient whose main symptom was masticatory disturbance associated with an extreme reduction in bite force.

## Case presentation

**Figure 1:**
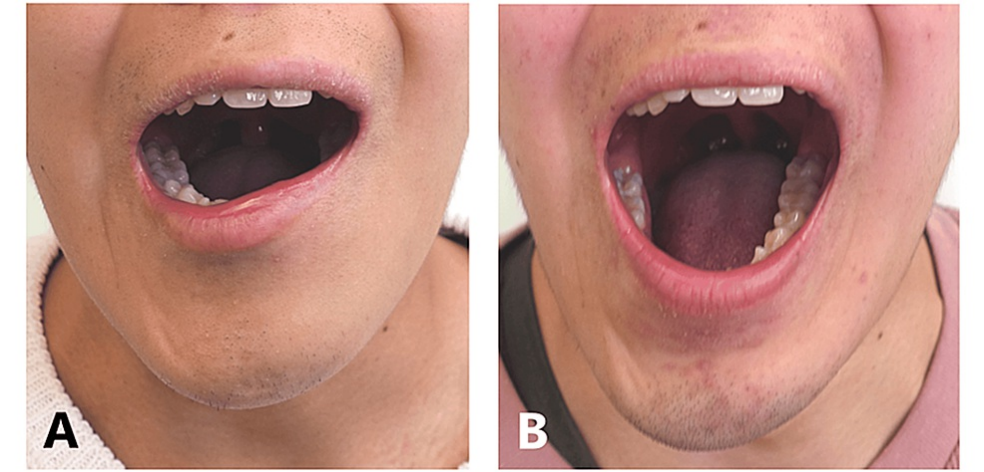
Front view of the patient’s mouth at maximum opening. At the first visit (35 days after disease onset), the patient’s mouth could open by just 24 mm (A). At one year and five months, mouth opening had improved to 40 mm (B).

Thirty-seven days before presenting to our department, a 17-year-old male high school student experienced a fever of 38°C and subsequently developed difficulty opening his mouth a few days later. The fever resolved after a week; however, the masticatory impairment persisted. Five days later, he visited the Department of Internal Medicine where a computed tomography scan was performed. However, no obvious finding suggestive of an inflammatory process was observed even after laboratory investigations. A non-steroidal anti-inflammatory drug (Celecoxib) was prescribed for jaw muscle pain. He also visited the Department of Otolaryngology and no obvious abnormality was found in his pharynx or larynx. The next day, he was referred to the Department of Oral and Maxillofacial Surgery. A dental examination showed that his temporomandibular joints were normal, and the cause of the masticatory disturbance was unknown. The patient was then referred to the Department of Neurology. The neurologist referred the patient to our hospital on suspicion of involuntary movements of the mandible or inflammation around the temporomandibular joints. His chief complaint was masticatory dysfunction due to extreme weakness of the muscles of mastication. After the fever had resolved, mouth opening became difficult, and his bite force had reduced. It became impossible to bite as he had to lift his mandible with his hands. He had bilateral tenderness of the masseter, temporalis, and lateral pterygoid muscles. His maximal mouth opening was 24 mm without jaw deviation, and asymmetry of the lower lip was observed (Figure 1, Panel A).

There were no clinical signs of focal involuntary movements suggestive of oromandibular dystonia. The possibility of inflammation around the temporomandibular joints was also ruled out based on the results of laboratory investigations. A lumbar puncture is usually performed in patients with suspected GBS to rule out infectious diseases [6]. In this case, GBS was strongly suspected due to the history of sudden and extreme muscle weakness after a febrile illness; therefore, no lumbar puncture was performed.

Measurements of bite force and grip strength

**Figure 2:**
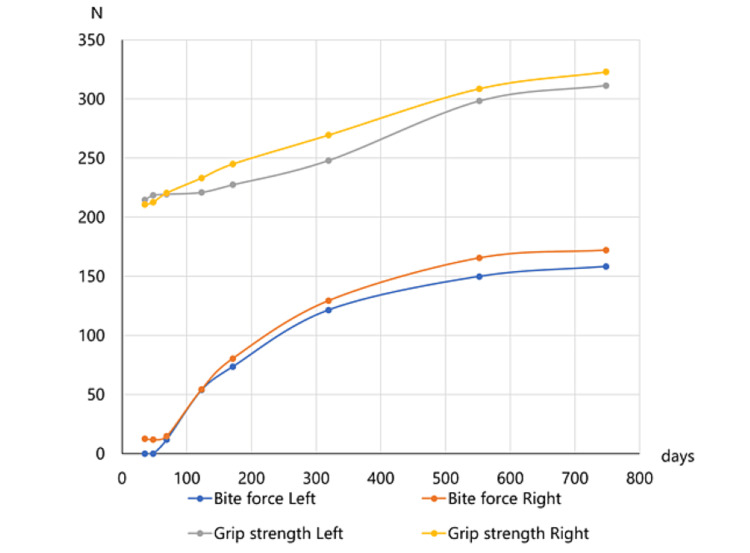
Changes in bite force and grip strength after the onset of Guillain-Barré syndrome. The patient’s bite force was extremely reduced; however, it gradually increased.

Maximum bite force and grip strength were measured to evaluate muscle weakness. The maximum bite force was measured on the bilateral first molars on three occasions using an occlusal force meter (GM10, Nagano Keiki Co.; Tokyo, Japan) [7]. At the first visit, it was impossible to measure bite force on the left side because a bite force could not be exerted at all, and the maximum bite force on the right side was 12.7 N (Figure 2).

The grip strength of both of the patient’s hands was recorded using a grip strength meter (ST3 T1781, Toei Light; Tokyo, Japan). The patient’s bite force and grip strength were measured at each visit (Figure 2).

Neurophysiological study

**Figure 3:**
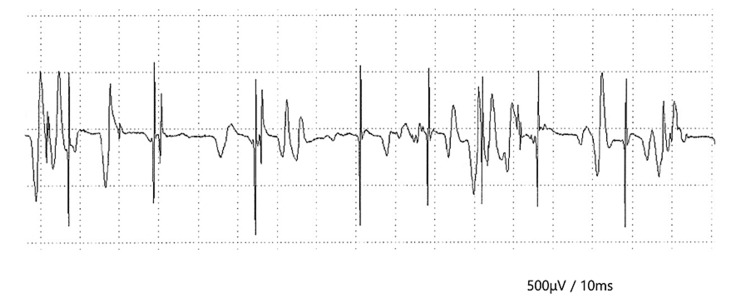
Masseter muscle electromyography using a needle electrode. Active denervation, late recruitment, and increased polyphasic units are observed.

A detailed neurophysiological study was performed by a neurologist 69 days after the onset of GBS. The ciliary sign was positive, and the jaw-jerk reflex was slightly enhanced. Vocalization was normal, and no dysphagia was observed. Nystagmus was also observed and limb reflexes were also slightly increased. Electrophysiological testing included a motor nerve conduction study (median, tibial, and facial nerves); a sensory nerve conduction study (median and sural nerves); F-waves of the median, tibial, and peroneal nerves; blink reflex; and needle electromyography of the masseter muscle using an electromyography instrument (MEB-2312, Nihon Kohden, Tokyo, Japan). The amplitude of the facial nerve impulses decreased with left stimulation, and the ratio of electrocorticography was 0.2 in the philtrum. The duration of the F-wave in the right tibial nerve was also prolonged. An H-wave was observed during the F-wave examination of the left tibial nerve; however, the blink reflex was normal. Active denervation and late recruitment of the left masseter muscle were observed on needle electromyography. Furthermore, the number of polyphasic units increased, and axonal disorders were observed (Figure 3).

Based on these findings, the patient was diagnosed with acute motor axonal neuropathy.

The patient’s symptoms were mild, and the chief complaint was masticatory disturbance. There was no difficulty in walking, breathing, or swallowing; therefore, the patient was carefully followed up at the outpatient clinic. The patient’s first visit to our hospital was 35 days after the onset of GBS. The disease course after onset was long, and typical treatments [8], such as intravenous immunoglobulin and plasma exchange, were not indicated. The patient’s condition was closely monitored during rehabilitation, which involved mouth opening, mastication training, and oral administration of vitamin B-12. The mouth opening training consisted of three sessions, held three times a day in the morning, afternoon, and evening [9]. Each session included passive mouth opening of approximately 40 mm manually or using a jaw-opening device (Heister’s jaw opener). The mouth opening distance was gradually increased, taking several minutes with a pause [10]. For mastication training, the patient was instructed to chew each bite of food 30 times.

The patient’s bite force and grip strength were both recorded at each visit (Figure 2). The decrease in muscle force was much more prominent for bite force than for grip strength (Figure 2). In particular, the bite force on the left side was not measured at the first and second visits because it was too low (Figure 2). The bilateral bite force and grip strength of both hands gradually improved over time (Figure 2). One year and five months after the disease onset, his maximal mouth opening was 40 mm, and the asymmetry of the lower lip had disappeared (Figure 1, Panel B). Subjective symptoms, such as masticatory disturbances, disappeared. The average bite force on both sides at the first visit (6.37 N) increased at the last visit to 165.3 N, which was 25.8 times higher (Figure 2). On the other hand, the average grip strength of both hands (212.7 N) increased to 317.2 N, representing a 1.5-fold increase (Figure 2).

## Discussion

To my knowledge, this case report is the first to describe changes in bite force in a patient with GBS who complained of severe masticatory disturbances due to extreme weakness of the masticatory muscles.

The estimated incidence of GBS in Western countries ranges from 0.89 to 1.89 cases per 100,000 person-years, and an increase of 20% is observed with every 10-year rise in age after the first decade of life [3]. GBS can develop within one to three weeks after a respiratory or gastrointestinal tract infection with several commonly found pathogens, including viruses and bacteria. These pathogens include *Campylobacter jejuni*, which is involved in approximately 30% of GBS cases and is a common cause of bacterial gastroenteritis; *Mycoplasma pneumoniae*; hepatitis E virus; cytomegalovirus; Epstein-Barr virus; and Zika virus [11-13]. The patient in this case report also experienced a fever of 38°C preceding the onset of his symptoms; however, the causative pathogen was not identified. GBS usually presents with symmetric ascending weakness and hyporeflexia, and weakness usually begins in the lower extremities [2]. The progression of the disease can be rapid, and most patients with GBS reach their maximum disability within two weeks [2]. The patient in this report also experienced slight lower limb weakness, which resolved considerably earlier than did the weakness of the masticatory muscles. Approximately 20% of patients with GBS develop respiratory failure and require mechanical ventilation. This involvement contributes to the estimated mortality rate of 3-10% in patients with GBS [13]. The chief complaint of the patient in this study was masticatory disturbance. During his first visit, it was impossible to measure the bite force on the left side because the jaw-closing muscles were extremely weakened. After a year and five months, the masticatory disturbance resolved, his maximum bite force was 149.9 N on the left side, and 165.6 N on the right side. His original bite force and grip strength before the onset of GBS are unknown; however, the patient’s height was 158.4 cm and he weighed 50 kg, which is considerably smaller than the average height of 170.6 cm for a 17-year-old Japanese man [14]. In addition, it is estimated that the grip strength was lower than the average grip strength of approximately 400 N for a 17-year-old Japanese man [15]. The decrease in bite force or masticatory disturbance in the patient with GBS had received no attention before the presentation at our department. In fact, the neurologist who referred the patient to the Department of Oral and Maxillofacial Surgery did not recognize that the patient had GBS and suspected involuntary mandibular movements. There are few reports on masticatory muscle weakness in patients with GBS. Pal and Sanyal [16] evaluated jaw muscle weakness in 24 patients with GBS. They assessed jaw-opening muscle weakness by requesting the patient to open their mouth against resistance from the examiner. They examined jaw-closing muscle weakness by asking the patients to close their jaws against a tongue spatula covered with gauze, which the examiner pulled against. Jaw-closing weakness was not observed in any of the 24 patients, whereas only one (4.2%) patient had a mild jaw-opening weakness. In contrast, muscle weakness was observed in adjoining muscles, such as the facial and neck flexor muscles, in 10 (41.7%) and 19 (79.2%) patients, respectively. They concluded patients with GBS very rarely had jaw muscle weakness [16].

The diagnosis of GBS is based on the patient’s history and neurological, electrophysiological, and cerebrospinal fluid examinations [7]. The diagnostic criteria for GBS were revised in 1990 by the National Institute of Neurological Disorders and Stroke [7]. The results of laboratory investigations can be used to exclude other causes of acute flaccid paralysis, such as infections or metabolic or electrolyte dysfunctions [2]. The classic finding in GBS is the combination of an elevated cerebrospinal fluid protein level and a normal cerebrospinal fluid cell count (known as albuminocytological dissociation) [1]. Albuminocytological dissociation is present in no more than 50% of patients with GBS during the first week of illness, although this percentage increases to 75% in the third week [17]. Cerebrospinal fluid examination was not considered for the patient in this report because of his mild symptoms. Nerve conduction studies can also help to support the diagnosis of GBS, as it can be used to distinguish between axonal and demyelinating subtypes [18].

GBS can be divided into at least four main subtypes, namely, acute inflammatory demyelinating polyradiculoneuropathy; the axonal subtypes, i.e., acute motor axonal neuropathy and acute motor and sensory axonal neuropathy; and Miller Fisher syndrome, the main symptoms of which are oculomotor dysfunction, ataxia, and areflexia [2]. In North America and Europe, approximately 5% of patients with GBS have the axonal subtypes, whereas in Central and South America, Japan, and China axonal subtypes account for approximately 30-47% of cases; Miller Fisher syndrome has been found to account for approximately 5% of cases of GBS [4]. Electrophysiological studies can distinguish between the subtypes of GBS [18]. Abnormal findings of electrophysiologic study in GBS have been previously described in detail [19]. The patient in this study was diagnosed with acute motor axonal neuropathy following electrophysiologic studies.

Early initiation of intravenous immunoglobulins or plasma exchange has been shown to be beneficial and crucial, especially in patients with rapidly progressive weakness. Plasma exchange appears to be most effective when it is started within the first two weeks after disease onset in patients who are unable to walk [6]. Treatment with intravenous immunoglobulin, initiated within two weeks after disease onset, is reported to be as effective as plasma exchange in patients with GBS who cannot walk independently [8]. In this case, the disease course after onset was long; therefore, intravenous immunoglobulin and plasma exchange were not indicated. The patient’s condition was closely monitored during rehabilitation, which included mouth opening training, mastication training, and oral administration of vitamin B12. However, the effects of mouth opening and mastication training on masticatory muscle weakness remain unclear. Only one randomized controlled trial has evaluated the efficacy of the proprioceptive neuromuscular facilitation technique on pulmonary function and diaphragmatic muscle activity in patients with GBS [20]. Vidhyadhari et al. evaluated 30 patients with GBS [20]. In the study, the experimental group (15 patients) performed repeated stabilization and rhythmic contraction, in addition to diaphragmatic breathing exercises, with three repetitions, three sets, and seven days in one week [20]. The control group (15 patients) performed diaphragmatic breathing exercises with the same volume, intensity, and frequency. Seven days later, significant differences in the parameters were found between the two groups; however, greater changes were observed in the experimental group than in the control group. The results demonstrate that proprioceptive neuromuscular facilitation techniques effectively enhance diaphragm muscle activity [20]. It is postulated that mouth opening and mastication training may hasten the recovery of masticatory muscle weakness in patients with GBS. However, further studies in sufficient cases may be needed.

## Conclusions

A 17-year-old male patient developed severe masticatory disturbance due to a significant reduction in bite force after a fever of 38°C. The patient was diagnosed with GBS (acute motor axonal neuropathy) after an electrophysiological study. The patient underwent rehabilitation comprising mouth opening and mastication training. The impairment was fully resolved at one year and five months.
